# Higher Urinary Iron Levels Are Associated with Kidney Dysfunction, Tubular Damage, and Increased Mortality in Kidney Transplant Recipients

**DOI:** 10.34067/KID.0000000878

**Published:** 2025-06-26

**Authors:** Daan Kremer, Pien Rawee, Tim J. Knobbe, Joanna Sophia J. Vinke, Kai Lüersen, David E. Leaf, Dorine W. Swinkels, Martin H. de Borst, Gerald Rimbach, Stephan J.L. Bakker, Michele F. Eisenga

**Affiliations:** 1 Division of Nephrology, Department of Internal Medicine, University of Groningen, University Medical Center Groningen, Groningen, The Netherlands; 2 Institute of Human Nutrition and Food Science, University of Kiel, Kiel, Germany; 3 Division of Renal Medicine, Brigham and Women's Hospital, Boston, Massachusetts; 4 Department of Laboratory Medicine, Radboud University Medical Center, Nijmegen, The Netherlands; 5 Sanquin Blood Bank, Amsterdam, The Netherlands

**Keywords:** kidney transplantation

## Abstract

**Key Points:**

- Kidney transplant recipients have higher urinary iron levels than controls and urinary iron levels are increased with oral iron supplementation.
- Urinary iron levels are associated with worse kidney function, increased tubular damage markers, and a higher mortality risk during follow-up.
- Further studies are warranted to provide insight into the potential tubulotoxic effect of urinary iron.

**Background:**

Increased urinary iron can result from (*1*) increased delivery of iron to the kidneys, (*2*) increased glomerular passage of iron, and/or (*3*) decreased tubular reuptake. Currently, the relevance of urinary iron levels is unknown. We investigated urinary iron with different pathways and clinical outcomes in kidney transplant recipient (KTRs).

**Methods:**

We measured urinary iron in samples from the prospective TransplantLines Food and Nutrition Biobank and Cohort study (NCT02811835). Multivariable linear and Cox regression models were applied.

**Results:**

We included 693 stable KTRs (age 53±13 years, 43% female, eGFR 52±20 ml/min per 1.73 m^2^). Higher urinary iron was associated with lower eGFR and higher kidney damage markers, including albuminuria, 24-hour urinary liver-type fatty acid-binding protein excretion, urinary endothelial growth factor to creatinine ratio, and plasma neutrophil-gelatinase–associated lipocalin (all *P* < 0.001). By contrast, urinary iron was not associated with systemic iron status but was increased with oral iron supplementation. During a follow-up of 5.3 years, 83 KTRs experienced graft failure and 150 died. Prospectively, higher urinary iron was associated with graft failure, but the association was decreased after adjustment for proteinuria. By contrast, urinary iron was independently associated with increased mortality risk (hazard ratio per doubling, 1.29; 95% confidence interval, 1.08 to 1.56).

**Conclusions:**

Higher urinary iron levels are associated with worse kidney function, more proteinuria, increased tubular damage markers and higher mortality. Oral iron supplementation seems to be an important determinant of urinary iron levels. These findings raise the possibility that urinary iron acts as a tubulotoxic agent, and mechanistic studies are warranted.

## Visual Abstract

**Figure:**
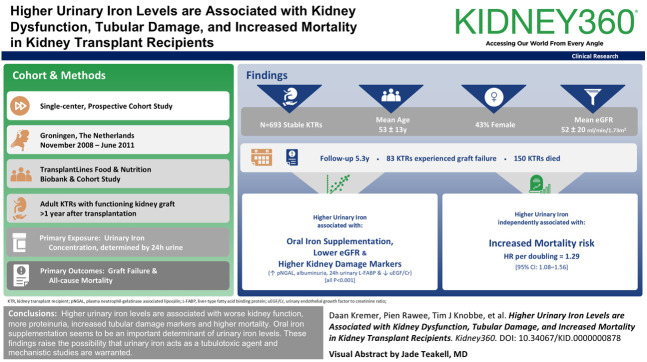

## Introduction

In the past decades, short-term outcomes after kidney transplantation have substantially improved. However, long-term graft and patient survival remain limited, emphasizing the need for better and earlier identification of kidney transplant recipients (KTRs) at an increased risk of adverse outcomes.^1^ Recently, the importance of an adequate iron status among KTRs has become increasingly clear.^2,3^

Iron homeostasis in this vulnerable patient group differs substantially from the general population due to changes in erythropoietin production and consequent increased iron utilization after transplantation, decreased iron absorption (presumably due to increased hepcidin concentrations), and intracellular iron shifts.^4^ As a result, iron deficiency is common among KTRs and is associated with adverse outcomes independent of anemia.^2,3^ In addition, some KTRs receive iron supplementation. To date, research among KTRs has primarily focused on concentrations of iron status parameters in blood. By contrast, the relevance of urinary iron has been investigated only to a minimal extent, although it represents an important part of iron homeostasis.^5^

Under physiologic circumstances, most filtered iron is reabsorbed in the proximal tubule.^6^ Several mechanisms might contribute to increased urinary iron concentrations. First of all, increased delivery of iron to the kidneys, *i.e*., when circulating iron levels are high, might result in higher urinary iron levels. Furthermore, increased glomerular passage of iron due to glomerular damage and/or decreased tubular reuptake of iron can contribute to the urinary iron concentration. Thus, increased urinary iron levels can be a marker of several pathophysiologic mechanisms. We hypothesize that increased urinary iron levels are associated with worse kidney function.

This study was primarily initiated to examine the potential relevance of urinary iron, with the following aims: (*1*) to identify determinants of urinary iron concentrations; (*2*) to investigate potential associations with kidney function and tubular damage markers; and (*3*) to assess putative prospective associations of urinary iron concentrations with graft failure and risk of death in KTRs.

## Methods

This study was performed in accordance with the guidelines for STrengthening the Reporting of OBservational studies in Epidemiology (see Supplemental Material for the checklist).^7^

### Study Population

**Figure 1:**
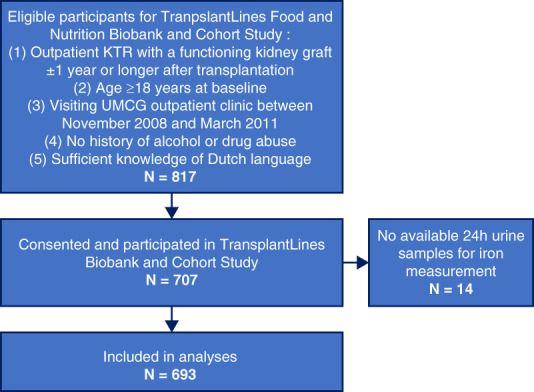
**Consolidated Standards of Reporting Trials diagram visualizing the flow of participants through the study.** KTR, kidney transplant recipient; UMCG, university medical center groningen.

For this study, we included adult KTRs participating in the previously described TransplantLines Food and Nutrition Biobank and Cohort study (NCT02811835).^8^ This single-center prospective cohort study included KTRs with a functioning kidney graft at least 1 year after transplantation visiting the outpatient clinic of the University Medical Center Groningen (Groningen, The Netherlands) between November 2008 and June 2011. The primary exposure was urinary iron concentration, determined in 693 KTRs with available 24-hour urine samples, which involves 98% of the total study population of 707 KTRs that were initially included in the cohort. All patients provided written informed consent. The study was approved by the University Medical Center Groningen Institutional Review Board (METc 2008/186) and adheres to the Declarations of Helsinki. The clinical and research activities being reported are consistent with the Principles of the Declaration of Istanbul as outlined in the “Declaration of Istanbul on Organ Trafficking and Transplant Tourism.” Follow-up was conducted using the continuous outpatient surveillance system. No patients were lost to follow-up. An overview of the flow of study participants is shown in Figure 1.

### Outcome Definitions

Primary outcomes of the study were graft failure and all-cause mortality. Graft failure was defined as return to dialysis or retransplantation and was censored for death. Cross-sectional secondary outcomes were markers of systemic iron status (iron, transferrin, ferritin, and transferrin saturation), kidney function (eGFR^9^, creatinine clearance), markers of tissue fibrosis (plasma endotrophin^10^), and markers of tubular damage (24-hour urinary liver-type fatty acid-binding protein excretion [uL-FABP]^11^, urinary endothelial growth factor to creatinine ratio [uEGF/Cr],^12^ and plasma neutrophil-gelatinase–associated lipocalin [pNGAL]^13^).

### Biochemical Analyses

Venous blood was drawn on the morning of the study visit after an overnight fasting period of at least 8 hours. In addition, patients were asked to collect urine during 24 hours before the study visit, according to a strict protocol.^14^ Samples were stored at −80°C until measurement. Urinary iron concentrations were measured using inductively coupled plasma mass spectrometry. A summary of analytical methods is presented in Supplemental Table 1. Iron deficiency was defined as a transferrin saturation <20% and ferritin <100 *µ*g/L.^15–17^ Patients were considered to have iron overload if the transferrin saturation was >45%.^18,19^

Urinary protein excretion was measured using the Biuret reaction (MEGA AU 150, Merck Diagnostica, Darmstadt, Germany). Other clinical chemistry assays, including hemoglobin, creatinine, inflammatory parameters (including high sensitivity C-reactive protein and leukocyte count), glucose, iron, ferritin, and transferrin, were performed using routine laboratory methods (Roche Diagnostics, Basel, Switzerland). In addition, uL-FABP and uEGF/Cr were determined using an ELISA, while pNGAL was measured using a particle-enhanced turbidimetric immunoassay, as described in detail elsewhere.^11–13^ Plasma endotrophin (PRO-C6) was measured using an ELISA (Nordic Bioscience, Herlev, Denmark), as described previously.^10^ Urinary nitrite (NO_2_^−^) and nitrate (NO_3_^−^) were measured by high-performance liquid chromatography.

### Statistical Analyses

Population characteristics at baseline are presented as mean±SD, median (interquartile range), or count (%) for normally distributed continuous variables, non-normally distributed continuous variables, and categorical variables, respectively. Univariable linear regression was used to identify associations between urinary iron concentrations and clinical and biochemical parameters. To identify potential determinants of urinary iron independent of age, sex, and eGFR, linear regression analyses were repeated with adjustment for these variables. All associations were assessed for fulfilment of the assumptions of linear regression. Variables were log_2_ transformed if necessary to fulfill these assumptions. To visualize the associations of urinary protein excretion with parameters of iron status and tubular damage, scatter plots with regression lines were plotted, and Pearson R^2^ is presented to quantify the proportion of variation in the respective variables explained by urinary iron concentration. Kaplan-Meier curves with risk tables were generated for the prospective outcomes, *i.e*., graft failure and mortality (Supplemental Figure 1). In prospective analyses, we studied the association of urinary iron concentration with time to graft failure and death using multivariable Cox models. These models were unadjusted (model 1) and adjusted for predefined variables including age, sex, time after transplantation (model 2), plus eGFR (model 3), 24-hour urinary protein excretion (model 4) or 24-hour urinary albumin excretion (model 5), and pre-emptive transplantation, history of cardiovascular disease, systolic BP, donor type (living or postmortal), and history of rejection (model 6). In addition to the variables included in model 4 to avoid risk of overfitting, we also adjusted for parameters of tubular damage and tissue fibrosis (model 7).^20^ Added prognostic value was assessed using the likelihood ratio test, comparing model 4 versus model 4 without urinary iron concentration. We assessed effect modification by age, sex, eGFR, and use of oral iron supplementation by adding interaction terms to the models. Moreover, potential nonlinear associations of urinary iron concentration with graft failure and mortality were assessed by adding restricted cubic spline terms (df=2 and df=3) to the final models. These were then compared with the models including log_2_ urinary iron concentration as a linear variable using likelihood ratio tests. The association between urinary iron concentration and graft failure and mortality is presented with 95% confidence intervals (CIs). All Cox proportional hazards models fulfilled the criterion of proportionality of hazards, as assessed by visual assessment of the Schoenfeld residuals. To account for missing data, multiple imputation was performed using fully conditional specification to obtain ten imputed datasets. The algorithm was run for ten iterations and convergence of the Markov chains was evaluated with trace plots of means and variances. The results were pooled using Rubin's rules. In sensitivity analyses, we repeated our analyses with exclusion of oral iron supplement users, with exclusion of patients with iron overload and with exclusion of patients with suspected urinary tract infection (UTI) based on a urinary nitrite (*μ*M)/nitrate (mM) ratio >130.^21^ In further sensitivity analyses, we repeated all analyses with 24-hour urinary iron excretion, rather than 24-hour urinary iron concentration. In secondary analyses, we assessed the association between urinary iron and cardiovascular and noncardiovascular mortality.

## Results

### Baseline Characteristics

In total, 693 KTRs were included in this study at 5.4 (2.0–12.0) years after transplantation. In this population, 299 participants (43%) were female, age was 53±13 years, and eGFR was 52±20 ml/min per 1.73 m^2^. The median urinary iron concentration was 14.1 (9.9–21.6) *µ*g/L. The median ferritin concentrations were 118 (55–221) *µ*g/L, and the mean transferrin saturation was 25.4%±10.8%. Oral iron supplementation was used by 41 (6%) KTRs, while none of the KTRs received intravenous iron supplementation. Additional baseline characteristics are presented in Table 1.

**Table 1:** Baseline characteristics, univariable, and multivariable linear regression analyses with log_2_ urinary iron concentration as the dependent variable

Variables	*N*=693	Linear Regression with log_2_ Urinary Iron Concentration as Dependent Variable		Linear Regression with log_2_ Urinary Iron Concentration as Dependent Variable Adj. for Age, Sex, and eGFR	
Urinary Iron Concentration (*µ*g/L)	14.1 (9.9 to 21.6)	St. *β* (95% CI)	*P* Value	St. *β* (95% CI)	*P* Value
**Clinical characteristics**					
Female sex, *n* (%)	299 (43)	−0.16 (−0.31 to −0.01)	0.033	−0.19 (−0.34 to −0.04)	0.097
Age, yr	53 (13)	−0.10 (−0.18 to −0.03)	0.008	−0.12 (−0.19 to−0.04)	0.002
Systolic BP, mm Hg	136 (18)	0.10 (0.03 to 0.18)	0.007	0.09 (0.02 to 0.17)	0.015
Diabetes, *n* (%)	162 (23)	−0.05 (−0.23 to 0.12)	0.55	0.01 (−0.17 to 0.18)	0.95
History of cardiovascular disease, *n* (%)	171 (25)	−0.11 (−0.28 to 0.07)	0.23	−0.07 (−0.25 to 0.10)	0.42
Smoking status, *n* (%)					
*Never*	273 (42)	Ref.		Ref.	
*History of smoking*	298 (46)	−0.01 (−0.18 to 0.15)	0.87	0.03 (−0.14 to 0.19)	0.74
*Current smoking*	81 (12)	0.24 (−0.00 to 0.49)	0.051	0.22 (−0.02 to 0.47)	0.070
Pre-emptive transplantation, *n* (%)	106 (15)	0.07 (−0.14 to 0.28)	0.52	0.05 (−0.16 to 0.26)	0.66
Time after transplantation, y^a^	5.4 (2.0–12.0)	−0.00 (−0.08 to 0.07)	0.99	0.03 (−0.04 to 0.10)	0.43
HLA class 2 antibodies, *n* (%)	121 (17)	0.24 (0.04 to 0.43)	0.019	0.21 (0.02 to 0.41)	0.033
History of rejection, *n* (%)	185 (27)	0.02 (−0.15 to 0.19)	0.84	−0.06 (−0.22 to 0.11)	0.52
History of delayed graft function, *n* (%)	52 (8)	0.26 (−0.02 to 0.55)	0.067	0.21 (−0.07 to 0.49)	0.15
Living donor, *n* (%)	242 (34)	0.04 (−0.12 to 0.20)	0.62	−0.00 (−0.17 to 0.16)	0.96
Donor age, yr	43 (15)	0.03 (−0.04 to 0.11)	0.41	−0.02 (−0.10 to 0.06)	0.65
**Other routine laboratory measurements**					
Sodium mmol/L	140.9 (2.8)	−0.04 (−0.12 to 0.03)	0.28	−0.03 (−0.11 to 0.04)	0.39
Potassium, mmol/L	4.0 (0.5)	0.16 (0.09 to 0.23)	<0.001	0.11 (0.04 to 0.19)	0.004
HbA1c, %^a^	5.8 (5.5–6.2)	−0.00 (−0.08 to 0.07)	0.91	0.04 (−0.01 to 0.13)	0.11
Leukocyte count, 10^9^/L	8.1 (2.6)	0.06 (−0.01 to 0.14)	0.096	0.06 (−0.01 to 0.13)	0.11
hs-CRP, mg/L^a^	1.6 (0.7–4.6)	0.03 (−0.05 to 0.11)	0.42	0.03 (−0.05 to 0.10)	0.46
AST, IU/L	24 (11)	0.03 (−0.05 to 0.10)	0.47	0.05 (−0.02 to 0.18)	0.11
ALT, IU/L	23 (18)	0.05 (−0.02 to 0.13)	0.17	0.07 (−0.01 to 0.14)	0.07
**Anemia and iron status parameters**					
Hemoglobin, g/dl	13.2 (1.8)	−0.12 (−0.19 to −0.05)	0.002	−0.09 (−0.18 to −0.01)	0.029
Mean corpuscular volume (fl)	90 (6)	0.03 (−0.08 to 0.14)	0.62	0.02 (−0.09 to 0.14)	0.68
Serum ferritin (*µ*g/L)^a^	118 (55–221)	0.09 (0.02 to 0.17)	0.018	0.08 (−0.00 to 0.15)	0.045
Serum iron (*µ*mol/L)	15 (11–19)	−0.01 (−0.09 to 0.06)	0.75	−0.01 (−0.09 to 0.06)	0.73
Transferrin (g/dl)	2.5 (0.4)	−0.09 (−0.16 to −0.01)	0.024	−0.06 (−0.14 to 0.01)	0.10
Transferrin saturation (%)	25.4 (10.8)	0.03 (−0.04 to 0.11)	0.40	0.02 (−0.05 to 0.10)	0.58
**Kidney function parameters**					
Creatinine, *µ*mol/L^a^	125 (100–161)	0.22 (−0.15 to 0.30)	<0.001	0.40 (0.12 to 0.68)	0.005
eGFR, ml/min per 1.73 m^2^	52 (20)	−0.16 (−0.23 to −0.09)	<0.001	−0.18 (−0.25 to −0.11)	<0.001
Urea, mmol/L^a^	9.6 (7.2–13.4)	0.18 (0.11 to 0.26)	<0.001	0.13 (0.01 to 0.26)	0.037
**Markers of glomerular/tubular damage and fibrosis**					
Urinary protein excretion, g/24 h^a^	0.2 (0.0–0.4)	0.41 (0.34 to 0.48)	<0.001	0.39 (0.32 to 0.46)	<0.001
Urinary liver-type fatty acid-binding protein, *µ*g/24 h^a^	2.1 (0.9–7.4)	0.33 (0.26 to 0.40)	<0.001	0.31 (0.22 to 0.39)	<0.001
Urinary endothelial growth factor/creatinine ratio, ng/mg^a^	6.4 (4.1–10.8)	−0.26 (−0.34 to −0.19)	<0.001	−0.22 (−0.33 to −0.10)	<0.001
pNGAL, *µ*g/L^a^	170 (134–232)	0.19 (0.12 to 0.27)	<0.001	0.11 (0.02 to 0.20)	0.020
Plasma endotrophin, ng/ml^a^	11.7 (9.2–13.3)	0.17 (0.09 to 0.24)	<0.001	0.09 (−0.01 to 0.18)	0.084
**Medication, *n* (%)**					
Oral iron supplementation	41 (6)	0.64 (0.33 to 0.95)	<0.001	0.54 (0.23 to 0.86)	0.001
Antihypertensive drugs	611 (88)	0.07 (−0.16 to 0.30)	0.53	0.01 (−0.22 to 0.25)	0.91
Prednisolone	686 (99)	0.52 (−0.23 to 1.26)	0.18	0.50 (−0.24 to 1.22)	0.19
Calcineurin inhibitor	397 (57)	0.01 (−0.14 to 0.16)	0.90	−0.11 (−0.27 to 0.04)	0.15
Proliferation inhibitor	573 (83)	0.01 (−0.18 to 0.21)	0.90	0.04 (−0.15 to 0.24)	0.66
mTOR inhibitor	24 (3)	0.04 (−0.37 to 0.45)	0.83	0.06 (−0.34 to 0.46)	0.76

Normally distributed data are presented as mean±SD, skewed data as median (interquartile range), and categorical data as number (valid percentage). eGFR as calculated using the creatinine-based CKD Epidemiology Collaboration formula^9^. ALT, alanine aminotransferase; AST, aspartate aminotransferase; CI, confidence interval; HbA1c, hemoglobin A1c; hs-CRP, high-sensitivity C-reactive protein; IU, international unit; mTOR, mammalian target of rapamycin; pNGAL, plasma neutrophil-gelatinase associated lipocalin.

a Variables were log_2_ transformed to fulfill the assumptions in linear regression analyses. Diabetes was defined according to the American Diabetes Association criteria. Data on smoking status were missing in 40 patients (6.0%), data on donor age were missing in 19 patients (2.8%), data on hemoglobin A1c were missing in 26 patients (3.9%), data on high-sensitivity C-reactive protein were missing in 39 patients (5.8%), data on urinary endothelial growth factor were missing in 52 patients (7.7%), and data on urinary liver-type fatty acid-binding protein were missing in 69 patients (10.1%). All other variables had missing data for ≤10 patients.

### Determinants of Urinary Iron

**Figure 2:**
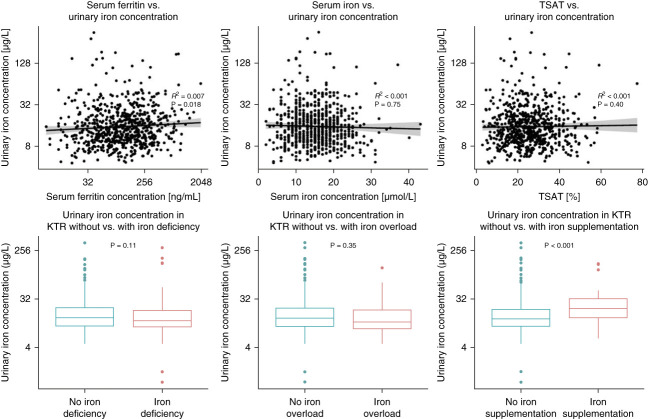
**Iron status parameters, iron status, and urinary iron concentration.** TSAT, transferrin saturation.

In univariable linear regression analyses, female sex, age, hemoglobin, transferrin, and eGFR were inversely associated with urinary iron concentrations (Table 1). Ferritin, systolic BP, presence of circulating HLA class 2 antibodies (irrespective of donor specificity), potassium, creatinine, urea, and use of oral iron supplementation were positively associated with urinary iron concentrations. In multivariable analyses, the associations of urinary iron with systolic BP, HLA class 2 antibodies, and iron supplementation remained independent of adjustment for age, sex, and eGFR. In addition, we found consistent associations between higher urinary iron concentration and kidney function parameters, including lower eGFR and higher plasma creatinine and urea (Table 1). The inverse associations of urinary iron with hemoglobin, and transferrin were no longer significant after adjustment for potential confounders. The association of iron status parameters and iron status is shown in Figure 2. Urinary iron concentration did not differ between patients with versus without iron overload or deficiency. However, oral iron supplement users did have higher urinary iron concentrations compared with those not using iron supplements.

### Associations of Urinary Iron with Markers of Kidney Damage

**Figure 3:**
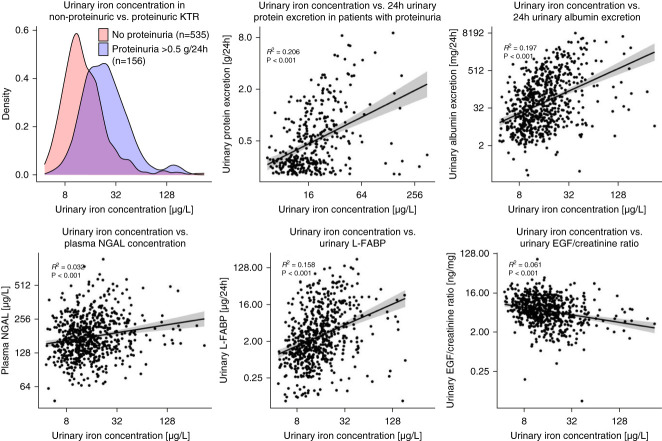
**Cross-sectional associations of urinary iron with parameters of glomerular/tubular damage.** EGF, endothelial growth factor; L-FABP, liver-type fatty acid-binding protein; NGAL, neutrophil-gelatinase associated lipocalin.

Interestingly, urinary iron was strongly associated with markers of tubular damage, including 24-hour urinary protein excretion. In fact, KTRs with and without proteinuria were found to have different urinary iron concentrations (Figure 3, A and B). In line with this, urinary iron was strongly associated with other tubular damage markers, including albuminuria, uL-FABP, uEGF/Cr, and pNGAL (Figure 3, C–F). The strong associations of urinary ion with urinary protein excretion, uL-FABP, and uEGF/Cr were independent of adjustment for age, sex, and eGFR. In addition, urinary iron was also positively associated with plasma endotrophin, a marker of tissue fibrosis, although this association was attenuated in multivariable analyses.

### Urinary Iron, Graft Failure, and Death

During a median follow-up of 5.3 (4.5–6.0) years, 83 patients (12%) experienced death-censored graft failure (Supplemental Figure 1). In unadjusted Cox regression analysis, urinary iron concentration was strongly associated with increased risk of graft failure (hazard ratio [HR], 1.65; 95% CI, 1.38 to 1.97). In multivariable Cox regression analysis, however, adjustment for 24-hour urinary protein excretion attenuated the association between urinary iron and graft failure such that it was no longer significant (Table 2).

**Table 2:** Univariable and adjusted Cox proportional hazards analyses for the association of urinary iron concentration with graft failure and mortality

Model	Graft Failure (*N*_events_=83, 12%)		Mortality (*N*_events_=150, 22%)	
	HR per Doubling (95% CI)	*P* Value	HR per Doubling (95% CI)	*P* Value
Model 1	1.65 (1.38 to 1.97)	<0.001	1.30 (1.12 to 1.51)	<0.001
Model 2	1.63 (1.36 to 1.95)	<0.001	1.45 (1.24 to 1.70)	<0.001
Model 3	1.35 (1.11 to 1.63)	0.003	1.38 (1.18 to 1.62)	<0.001
Model 4	1.08 (0.86 to 1.37)	0.5	1.28 (1.07 to 1.52)	0.006
Model 5	1.02 (0.80 to 1.32)	0.9	1.33 (1.12 to 1.58)	0.001
Model 6	1.20 (0.93 to 1.54)	0.16	1.30 (1.09 to 1.56)	0.004
Model 7	1.14 (0.89 to 1.47)	0.3	1.29 (1.08 to 1.56)	0.006

Model 1, univariable. Model 2, adjusted for age, sex, and log_2_ time after transplantation. Model 3, adjusted for variables in model 2+eGFR. Model 4, adjusted for variables in model 3+log_2_ 24 hours urinary protein excretion. Model 5, adjusted for variables in model 3+log_2_ 24 hours urinary albumin excretion. Model 6, adjusted for variables in model 4+pre-emptive transplantation, history of cardiovascular disease, systolic BP, donor type (living or postmortal) and history of rejection. Model 7, adjusted for variables in model 4+urinary EGF to creatinine ratio, plasma neutrophil gelatinase-associated lipocalin, 24 hours urinary liver-type fatty acid-binding protein, and plasma endotrophin. Addition of urinary iron concentration significantly improved the model fit for mortality (*P*_likelihood ratio_ = 0.008) but not for graft failure (*P*_likelihood ratio_ = 0.7). There was no evidence for nonlinearity (*P*_likelihood ratio_ = 0.9 and *P*_likelihood ratio_ = 0.8 for graft failure and mortality, respectively). CI, confidence interval; EGF, endothelial growth factor; HR, hazard ratio.

**Figure 4:**
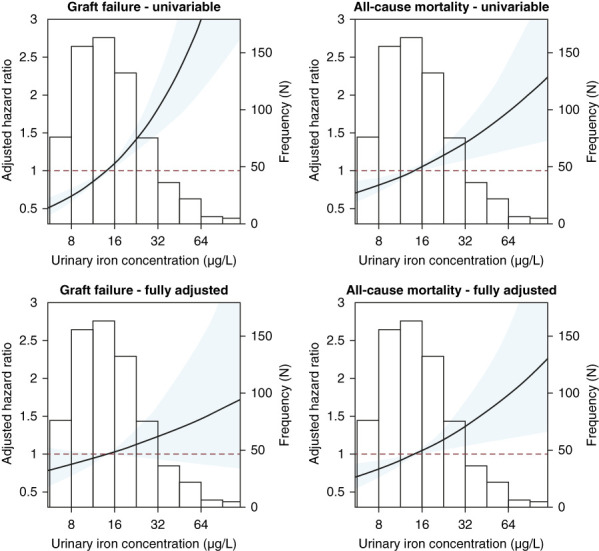
**Association between urinary iron excretion and graft failure and all-cause mortality.** Point estimates for the hazard ratio are represented by the black line. The shaded area represents the 95% CI. Hazard ratios are based on a Cox regression model, adjusted for age, sex, eGFR, log_2_ urinary protein excretion, log_2_ time after transplantation, pre-emptive transplantation, cardiovascular history, systolic BP, donor type (living or postmortal), and history of rejection. CI, confidence interval.

During a median follow-up of 5.4 (4.8–6.1) years, 150 KTRs (22%) died. In univariable Cox regression analysis, urinary iron was strongly associated with increased risk of death (HR, 1.30; 95% CI, 1.12 to 1.51). The association remained essentially unchanged after multivariable adjustment (HR, 1.30; 95% CI, 1.09 to 1.56; Figure 4 and Table 2).

### Sensitivity Analyses

The results of the Cox analyses remained materially unchanged when excluding patients with iron overload (*i.e*., transferrin saturation >45%; *N*=33; Supplemental Table 2), when excluding patients using iron supplementation (*N*=41; Supplemental Table 3) and when excluding patients with suspected UTI (*N*=66, Supplemental Table 4).

Similar results were also obtained when assessing 24-hour urinary iron excretion, rather than urinary iron concentration, as presented in Supplemental Table 5.

### Secondary Analyses—Urinary Iron and Cause-Specific Mortality

Urinary iron concentration was particularly strongly associated with cardiovascular mortality (HR, 1.42; 95% CI, 1.13 to 1.79; *P* = 0.003), whereas urinary iron was not significantly associated with mortality from other causes (HR, 1.21; 95% CI, 0.99 to 1.49; *P* = 0.063). This observation remained consistent on adjustment for potential confounders, as presented in Table 3.

**Table 3:** Univariable and adjusted Cox proportional hazards analyses for the association of urinary iron concentration with cause-specific mortality

Model	Cardiovascular Mortality (*N*_events_=60/693, 9%)		Noncardiovascular Mortality (*N*_events_=90/693, 13%)	
	HR per Doubling (95% CI)	*P* Value	HR per Doubling (95% CI)	*P* Value
Model 1	1.42 (1.13 to 1.79)	0.003	1.21 (0.99 to 1.49)	0.063
Model 2	1.57 (1.23 to 1.98)	<0.001	1.38 (1.12 to 1.70)	0.003
Model 3	1.47 (1.16 to 1.87)	0.002	1.32 (1.07 to 1.63)	0.012
Model 4	1.34 (1.03 to 1.76)	0.032	1.23 (0.97 to 1.56)	0.081

Model 1, univariable. Model 2, adjusted for age, sex, and log_2_ time after transplantation. Model 3, adjusted for variables in model 2+eGFR. Model 4, adjusted for variables in model 3+log_2_ 24 hours urinary protein excretion. Addition of urinary iron concentration significantly improved the model fit for cardiovascular mortality (*P*_likelihood ratio_ = 0.037) but not for noncardiovascular mortality (*P*_likelihood ratio_ = 0.088). Again, there was no evidence for effect modification by age, sex, eGFR or 24-hour urinary protein excretion. CI, confidence interval; HR, hazard ratio.

## Discussion

In this study, we show, in line with our hypothesis, that in KTRs urinary iron concentrations are strongly associated with proteinuria and markers of tubular damage. Urinary iron was not strongly associated with systemic iron status but was linked to oral iron supplementation. We identified a strong association between urinary iron and increased risk of death independent of kidney function, urinary protein excretion, cardiovascular parameters, and tubular damage markers.

**Figure 5:**
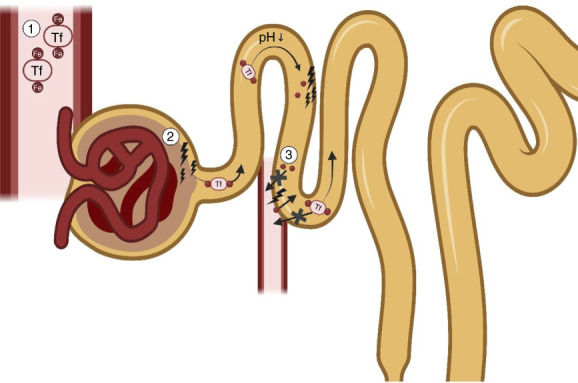
**Graphical representation of the hypothesized mechanisms contributing to urinary iron levels.** 1. Increased delivery of iron to the kidneys. 2. Increased glomerular passage of iron. 3. Decreased tubular uptake of iron and/or increased excretion. Created with Biorender.

The median urinary iron concentrations among KTRs in our study were around 14 *µ*g/L, which is multiple times higher compared with the estimated and reported urinary iron concentrations in healthy controls.^5,22^ Conceptually, there are two potential explanations of the increased urinary iron concentrations in KTRs. First, iron overload with consequent high circulating iron concentrations may theoretically lead to an increased glomerular filtration of iron, which may result in increased urinary iron concentrations (Figure 5).^5^ However, circulating iron concentrations were not a main determinant of urinary iron concentration, as evidenced by the limited associations of iron status parameters in blood with urinary iron concentrations. A previous study also showed that urinary iron levels were not related to urinary or serum hepcidin levels in patients with tubular dysfunction.^22^ We also did not expect iron status to be the main determinant, as iron deficiency rather than iron overload is common in KTRs. Notably, iron supplementation was strongly associated with higher urinary iron excretion in KTRs. It has previously been reported that the fast absorption of oral iron supplements (ferrous fumarate or ferrous sulfate in this cohort) creates a transient rise in nontransferrin bound iron (NTBI).^23,24^ NTBI may then be freely filtered by the kidneys, contributing to increased urinary iron levels.

The other potential explanation for the increased urinary iron concentrations in KTRs could be due to glomerular and tubular dysfunction (Figure 5). In general, glomerular and tubular damage are common among KTRs, as the single functional kidney in these patients has generally suffered from ischemia and oxidative stress during transplantation. The necessary use of nephrotoxic drugs including calcineurin inhibitors puts more strain to the kidney.

On the one hand, this acquired injury can result in leakage of the glomeruli, thus increasing the filtration of iron-carrying proteins.^5^ It is expected that a substantial portion of the KTRs have glomerular injury with loss of podocytes due to, *e.g*., diabetes or cardiovascular diseases. This is believed to account for a significant portion of the increased urinary iron levels.^5^

On the other hand, the kidney damage in KTRs can result in tubular damage and dysfunction. One of the key functions of the tubuli is the reabsorption of nutrients, including iron, from the tubular lumen. Under physiologic circumstances, the majority of iron is reabsorbed in the proximal tubule.^5^ Previously, it has already been found that in several different causes of CKD with glomerular dysfunction, iron deposition in the proximal tubuli exists.^6^ Tubular damage and dysfunction, particularly of the proximal tubule, can conceptually lead to decreased reabsorption of iron from the tubular lumen and consequently increased urinary iron concentrations. Indeed, our analyses suggest that tubular damage is a determinant of urinary iron concentrations, as evidenced by the strong associations of urinary iron concentrations with other markers of tubular damage, including liver-type fatty acid-binding protein excretion, urinary endothelial growth factor/creatinine concentration, and pNGAL. In addition, in a previous study, increased urinary iron levels were found in individuals with renal tubular dysfunction compared with healthy controls.^22^ Potentially, in addition to decreased iron reuptake due to tubular dysfunction, tubular damage with increased exfoliation of damaged tubular cells with concomitant increased release of iron in (pre-)urine may also explain the association between tubular damage markers and urinary iron.

The mechanism of urinary iron as a reflection of glomerular and tubular damage may also explain the association of urinary iron with graft failure and mortality. The association with graft failure appeared to be mediated by glomerular and tubular damage, as it disappears after additional adjustment for parameters of glomerular and tubular damage (also see Figure 4, fully adjusted model). The association between higher urinary iron concentrations and higher risk of mortality remained, independent of extensive adjustment for potential confounders. One may hypothesize that this association is a direct result of excessive iron loss, with consequent iron deficiency and detrimental effects on prognosis. However, this hypothesis appears unlikely because of the lack of an association between urinary iron concentrations and circulating iron status parameters. In addition, the urinary losses observed in our study are rather small relative to total body iron content.

Alternatively, the notion that high urinary iron reflects tubular dysfunction that contributes to cardiovascular morbidity appears a more likely explanation for the association with mortality. Urinary iron was particularly associated with cardiovascular mortality, even after correction for proteinuria and eGFR. Previously, tubular function markers have been found to be associated with cardiovascular events independently of eGFR and albuminuria (*i.e*. more glomerular markers of kidney function) in patients with nondiabetic CKD.^25^ This leads us to speculate that the underlying mechanism of the association between urinary iron and cardiovascular mortality could be the result of tubular dysfunction.

In addition to urinary iron as a tubular marker, our findings raise the hypothesis of iron as a tubulotoxic agent. The increased leakage of iron-carrying proteins (*e.g*., transferrin) due to glomerular damage and subsequent disassociation of iron from transferrin in a more acidic environment could potentially contribute to proximal tubular damage. In addition, the transient rise of NTBI after oral iron supplementation^1,2^ might contribute to iron levels in the tubular fluid as NTBI is freely filtered by the glomerulus. Unlike transferrin-bound iron, NTBI exists either in a free state or loosely bound to proteins, making it more susceptible to participating in redox reactions and causing oxidative damage in the tubules. Unfortunately, the data of this study cannot elucidate whether our hypothesis of iron acting as a tubulotoxic agent is true. There are data available that (primarily parenteral) iron formulations might contribute to kidney damage, presumably through oxidative stress and induction of tubular and endothelial cell death.^26–28^ However, evidence on the topic is sparse and ambiguous. Reassuringly, the largest randomized controlled trial involving nondialysis CKD patients (The Finerenone In Non-Diabetic Chronic Kidney Disease study) showed a lack of renal toxicity after intravenous iron administration in a 1-year follow-up.^29^ More research into this topic is warranted to further establish safe limits of iron supplementation and to provide mechanistic insights into possible detrimental effects of iron.^26,28,29^

A strength of this study is its large population size, with no loss to follow-up. In addition, the extensive availability of clinical and biochemical parameters allowed for an in-depth assessment of the potential meaning of urinary iron, as well as extensive adjustment for potential confounders. While the use of 24-hour urine samples can be considered a strength, as it accounts for fluctuations in iron excretion during the day, it also presents certain limitations. The collection process is burdensome for participants and can be suspectible to errors, such as missed voids. Given the observational study design, we can speculate regarding the role and meaning of urinary iron, but we cannot provide causal evidence. In addition, another limitation is that other tubular markers, *e.g*., kidney injury molecule-1, would have given additional information regarding proximal tubular damage. Furthermore, the absence of data on urine analysis is a limitation, as it would have improved the definition of those with suspected UTI in our sensitivity analysis. Moreover, future studies should assess the degree to which urinary iron is transferrin-bound or labile iron. Given the potential tubulotoxicity of labile iron, it would be relevant to distinguish between these two forms and study the association between transferrin-bound and labile iron with graft failure or mortality. Comparing NTBI levels before and after oral or intravenous iron supplementation can provide insight into factors contributing to NTBI levels. Additional preclinical and clinical studies are needed to further assess the hypotheses generated by this paper. Finally, the study population consisted mainly of White participants from The Netherlands. Ideally, the study should therefore be replicated in other areas to confirm its validity across different KTR populations.

In conclusion, urinary iron concentrations are strongly associated with markers of tubular and glomerular damage and graft failure. The latter association disappeared after adjustment for urinary protein excretion. By contrast, the association of urinary iron with a higher risk of mortality remained independent of adjustment for potential confounders. These data suggest that urinary iron concentration may be a hallmark of glomerular and tubular dysfunction and warrant further investigation into urinary iron as a potential tubulotoxic agent.

## Supplementary Material

## Data Availability

Partial restrictions to the data and/or materials apply. Public sharing of individual participant data was not included in the informed consent forms of the TransplantLines Biobank and Cohort Study, but data will be made available to interested researchers upon reasonable request after approval by the TransplantLines scientific committee (email: datarequest.transplantlines@umcg.nl). The data that support the findings of this study are available on request from the corresponding author. The data are not publicly available due to privacy or ethical restrictions.

## Declarative Statements

This study includes clinical experimentation and received Institutional Review Board or Ethics Committee approval. All patients provided written informed consent. This study includes clinical experimentation and complies with the Declaration of Helsinki. The clinical and research activities being reported are consistent with the Principles of the Declaration of Istanbul as outlined in the Declaration of Istanbul on Organ Trafficking and Transplant Tourism.

## Supplemental Material

Supplemental Figure 1. Kaplan-Meier curves for graft failure and mortality.

Supplemental Table 1. Iron analysis using inductively coupled plasma mass spectrometry.

Supplemental Table 2. Univariable and adjusted Cox proportional hazards analyses for the association of urinary iron concentration with graft failure and mortality, with exclusion of patients with iron overload (*i.e*., transferrin saturation >45%; *N*=33).

Supplemental Table 3. Univariable and adjusted Cox proportional hazards analyses for the association of urinary iron concentration with graft failure and mortality, with exclusion of patients using iron supplementation (*N*=41).

Supplemental Table 4. Univariable and adjusted Cox proportional hazards analyses for the association of urinary iron concentration with graft failure and mortality, with exclusion of patients with suspected UTI (*N*=66).

Supplemental Table 5. Univariable and adjusted Cox proportional hazards analyses for the association of 24-hour urinary iron excretion (rather than concentration) with graft failure and mortality.

Strobe Checklist. Checklist of items that should be included in reports of observational studies.
