# Supplementary material for: Higher Urinary Iron Levels Are Associated with Kidney Dysfunction, Tubular Damage, and Increased Mortality in Kidney Transplant Recipients
Source: Kidney360. 2025 Jun 26;6(11):1970–80. doi: 10.34067/KID.0000000878 (PMC12626665; doi:10.34067/KID.0000000878)
Supplement: Supplementary file 1 [file kidney360-6-1970-s001.pdf]

ASN American Society of Nephrology
JASN Journal of the American Society of Nephrology
KIDNEY360 Accessing Our World From Every Angle
CJASN Clinical Journal of the American Society of Nephrology

ASN Journal Disclosure Form

As per ASN journal policy, I have disclosed any financial relationships or commitments I have held in the past 36 months as included below. I have listed my Current Employer below to indicate there is a relationship requiring disclosure. If no relationship exists, my Current Employer is not listed.

S. Bakker reports the following:
Employer: University Medical Center Groningen; and Research Funding: Astellas Pharma, Chiesi, bioMerieux.

I understand that the information above will be published within the journal article, if accepted, and that failure to comply and/or to accurately and completely report the potential financial conflicts of interest could lead to the following: 1) Prior to publication, article rejection, or 2) Post-publication, sanctions ranging from, but not limited to, issuing a correction, reporting the inaccurate information to the authors' institution, banning authors from submitting work to ASN journals for varying lengths of time, and/or retraction of the published work.

Name: Stephan J.L. Bakker
Manuscript ID: K360-2025-000408R1
Manuscript Title: Higher Urinary Iron Levels are Associated with Kidney Dysfunction, Tubular Damage, and Increased Mortality in Kidney Transplant Recipients
Date of Completion: May 21, 2025
Disclosure Updated Date: May 9, 2025

ASN American Society of Nephrology JASN Journal of the American Society of Nephrology KIDNEY360 Accessing Our World From Every Angle CJASN Clinical Journal of the American Society of Nephrology

ASN Journal Disclosure Form

As per ASN journal policy, I have disclosed any financial relationships or commitments I have held in the past 36 months as included below. I have listed my Current Employer below to indicate there is a relationship requiring disclosure. If no relationship exists, my Current Employer is not listed.

M. De Borst reports the following:
Employer: University Medical Center Groningen, The Netherlands; Consultancy: Astellas; Astra Zeneca; Bayer; Boehringer Ingelheim; Lily; Novo Nordisk; Research Funding: CSL Vifor; and Advisory or Leadership Role: Theme Editor: Nephrology Dialysis Transplantation; Advisory Board: Lancet eClinicalMedicine; Board member of the ERA CKD-MBD Working Group; Member of the Dutch Kidney Foundation Scientific Council; Founder and Chair: NEFRO-NL.

I understand that the information above will be published within the journal article, if accepted, and that failure to comply and/or to accurately and completely report the potential financial conflicts of interest could lead to the following: 1) Prior to publication, article rejection, or 2) Post-publication, sanctions ranging from, but not limited to, issuing a correction, reporting the inaccurate information to the authors' institution, banning authors from submitting work to ASN journals for varying lengths of time, and/or retraction of the published work.

Name: Martin H. De Borst
Manuscript ID: K360-2025-000408R1
Manuscript Title: Higher Urinary Iron Levels are Associated with Kidney Dysfunction, Tubular Damage, and Increased Mortality in Kidney Transplant Recipients
Date of Completion: May 21, 2025
Disclosure Updated Date: May 20, 2025

ASN
American Society of Nephrology
JASN
Journal of the American Society of Nephrology
KIDNEY360
Accessing Our World From Every Angle
CJASN
Clinical Journal of the American Society of Nephrology

ASN Journal Disclosure Form

As per ASN journal policy, I have disclosed any financial relationships or commitments I have held in the past 36 months as included below. I have listed my Current Employer below to indicate there is a relationship requiring disclosure. If no relationship exists, my Current Employer is not listed.

M. Eisenga reports the following:
Employer: University Medical Center Groningen; Consultancy: Cablon Medical; Research Funding: Astellas; Cablon Medical; Advisory or Leadership Role: Cablon Medical; Medice; GSK; and Speakers Bureau: Astellas, GlaxoSmithKline, Medice, and Pharmacosmos.

I understand that the information above will be published within the journal article, if accepted, and that failure to comply and/or to accurately and completely report the potential financial conflicts of interest could lead to the following: 1) Prior to publication, article rejection, or 2) Post-publication, sanctions ranging from, but not limited to, issuing a correction, reporting the inaccurate information to the authors' institution, banning authors from submitting work to ASN journals for varying lengths of time, and/or retraction of the published work.

Name: Michele F. Eisenga
Manuscript ID: K360-2025-000408R1
Manuscript Title: Higher Urinary Iron Levels are Associated with Kidney Dysfunction, Tubular Damage, and Increased Mortality in Kidney Transplant Recipients
Date of Completion: May 22, 2025
Disclosure Updated Date: May 22, 2025

ASN American Society of Nephrology | JASN Journal of the American Society of Nephrology | KIDNEY360 Accessing Our World From Every Angle | CJASN Clinical Journal of the American Society of Nephrology

ASN Journal Disclosure Form

As per ASN journal policy, I have disclosed any financial relationships or commitments I have held in the past 36 months as included below. I have listed my Current Employer below to indicate there is a relationship requiring disclosure. If no relationship exists, my Current Employer is not listed.

T. Knobbe reports the following:
Employer: UMCG; and Research Funding: The TransplantLines Biobank and Cohort study was supported by a grant from Astellas BV and Chiesi Pharmaceuticals BV, and co-financed by the Dutch Ministry of Economic Affairs and Climate Policy by means of the PPP-allowance made available by the Top Sector Life Sciences & Health to stimulate public-private partnerships.

I understand that the information above will be published within the journal article, if accepted, and that failure to comply and/or to accurately and completely report the potential financial conflicts of interest could lead to the following: 1) Prior to publication, article rejection, or 2) Post-publication, sanctions ranging from, but not limited to, issuing a correction, reporting the inaccurate information to the authors' institution, banning authors from submitting work to ASN journals for varying lengths of time, and/or retraction of the published work.

Name: Tim J. Knobbe
Manuscript ID: K360-2025-000408R1
Manuscript Title: Higher Urinary Iron Levels are Associated with Kidney Dysfunction, Tubular Damage, and Increased Mortality in Kidney Transplant Recipients
Date of Completion: June 1, 2025
Disclosure Updated Date: June 1, 2025

ASN American Society of Nephrology
JASN Journal of the American Society of Nephrology
KIDNEY360 Accessing Our World From Every Angle
CJASN Clinical Journal of the American Society of Nephrology

ASN Journal Disclosure Form

As per ASN journal policy, I have disclosed any financial relationships or commitments I have held in the past 36 months as included below. I have listed my Current Employer below to indicate there is a relationship requiring disclosure. If no relationship exists, my Current Employer is not listed.

D. Kremer reports the following:
Employer: University of Groningen and University Medical Centre Groningen, Groningen, The Netherlands; and Research Funding: The TransplantLines Food and Nutrition Biobank and Cohort Study (ClinicalTrials.gov; #NCT02811835) was financially supported by Top Institute Food and Nutrition (grant A-1003). In addition, this collaboration project is co-financed by the Dutch Ministry of Economic Affairs and Climate Policy by means of the PPP-allowance made available by the Top Sector Life Sciences & Health to stimulate public-private partnerships.

I understand that the information above will be published within the journal article, if accepted, and that failure to comply and/or to accurately and completely report the potential financial conflicts of interest could lead to the following: 1) Prior to publication, article rejection, or 2) Post-publication, sanctions ranging from, but not limited to, issuing a correction, reporting the inaccurate information to the authors' institution, banning authors from submitting work to ASN journals for varying lengths of time, and/or retraction of the published work.

Name: Daan Kremer
Manuscript ID: K360-2025-000408R1
Manuscript Title: Higher Urinary Iron Levels are Associated with Kidney Dysfunction, Tubular Damage, and Increased Mortality in Kidney Transplant Recipients
Date of Completion: June 1, 2025
Disclosure Updated Date: June 1, 2025

ASN American Society of Nephrology
JASN Journal of the American Society of Nephrology
KIDNEY360 Accessing Our World From Every Angle
CJASN Clinical Journal of the American Society of Nephrology

ASN Journal Disclosure Form

As per ASN journal policy, I have disclosed any financial relationships or commitments I have held in the past 36 months as included below. I have listed my Current Employer below to indicate there is a relationship requiring disclosure. If no relationship exists, my Current Employer is not listed.

D. Leaf reports the following:
Consultancy: Sidereal Therapeutics, Casma Therapeutics, MexBrain, Entrada Therapeutics, CardioRenal Systems, Inc., and Alexion Pharmaceuticals; Research Funding: BioPorto, BTG International, Metro International Biotech LLC, Renibus Therapeutics, Inc., and Alexion Pharmaceuticals; and Advisory or Leadership Role: Scientific Advisory Board of CardioRenal Systems, Inc. (paid capacity).

I understand that the information above will be published within the journal article, if accepted, and that failure to comply and/or to accurately and completely report the potential financial conflicts of interest could lead to the following: 1) Prior to publication, article rejection, or 2) Post-publication, sanctions ranging from, but not limited to, issuing a correction, reporting the inaccurate information to the authors' institution, banning authors from submitting work to ASN journals for varying lengths of time, and/or retraction of the published work.

Name: David E. Leaf
Manuscript ID: K360-2025-000408R1
Manuscript Title: Higher Urinary Iron Levels are Associated with Kidney Dysfunction, Tubular Damage, and Increased Mortality in Kidney Transplant Recipients
Date of Completion: May 21, 2025
Disclosure Updated Date: November 11, 2024

ASN American Society of Nephrology
JASN Journal of the American Society of Nephrology
KIDNEY360 Accessing Our World From Every Angle
CJASN Clinical Journal of the American Society of Nephrology

ASN Journal Disclosure Form

As per ASN journal policy, I have disclosed any financial relationships or commitments I have held in the past 36 months as included below. I have listed my Current Employer below to indicate there is a relationship requiring disclosure. If no relationship exists, my Current Employer is not listed.

K. Luersen reports the following:
Employer: University of Kiel

I understand that the information above will be published within the journal article, if accepted, and that failure to comply and/or to accurately and completely report the potential financial conflicts of interest could lead to the following: 1) Prior to publication, article rejection, or 2) Post-publication, sanctions ranging from, but not limited to, issuing a correction, reporting the inaccurate information to the authors' institution, banning authors from submitting work to ASN journals for varying lengths of time, and/or retraction of the published work.

Name: Kai Luersen
Manuscript ID: K360-2025-000408R1
Manuscript Title: Higher Urinary Iron Levels are Associated with Kidney Dysfunction, Tubular Damage, and Increased Mortality in Kidney Transplant Recipients
Date of Completion: May 22, 2025
Disclosure Updated Date: May 22, 2025

ASN American Society of Nephrology JASN Journal of the American Society of Nephrology KIDNEY360 Accessing Our World From Every Angle CJASN Clinical Journal of the American Society of Nephrology

ASN Journal Disclosure Form

As per ASN journal policy, I have disclosed any financial relationships or commitments I have held in the past 36 months as included below. I have listed my Current Employer below to indicate there is a relationship requiring disclosure. If no relationship exists, my Current Employer is not listed.

P. Rawee has nothing to disclose.

I understand that the information above will be published within the journal article, if accepted, and that failure to comply and/or to accurately and completely report the potential financial conflicts of interest could lead to the following: 1) Prior to publication, article rejection, or 2) Post-publication, sanctions ranging from, but not limited to, issuing a correction, reporting the inaccurate information to the authors' institution, banning authors from submitting work to ASN journals for varying lengths of time, and/or retraction of the published work.

Name: Pien Rawee
Manuscript ID: K360-2025-000408R1
Manuscript Title: Higher Urinary Iron Levels are Associated with Kidney Dysfunction, Tubular Damage, and Increased Mortality in Kidney Transplant Recipients
Date of Completion: June 5, 2025
Disclosure Updated Date: June 5, 2025

ASN American Society of Nephrology
JASN Journal of the American Society of Nephrology
KIDNEY360 Accessing Our World From Every Angle
CJASN Clinical Journal of the American Society of Nephrology

ASN Journal Disclosure Form

As per ASN journal policy, I have disclosed any financial relationships or commitments I have held in the past 36 months as included below. I have listed my Current Employer below to indicate there is a relationship requiring disclosure. If no relationship exists, my Current Employer is not listed.

G. Rimbach reports the following:
Employer: University of Kiel

I understand that the information above will be published within the journal article, if accepted, and that failure to comply and/or to accurately and completely report the potential financial conflicts of interest could lead to the following: 1) Prior to publication, article rejection, or 2) Post-publication, sanctions ranging from, but not limited to, issuing a correction, reporting the inaccurate information to the authors' institution, banning authors from submitting work to ASN journals for varying lengths of time, and/or retraction of the published work.

Name: Gerald Rimbach
Manuscript ID: K360-2025-000408R1
Manuscript Title: Higher urinary iron levels...
Date of Completion: May 21, 2025
Disclosure Updated Date: May 21, 2025

ASN American Society of Nephrology JASN Journal of the American Society of Nephrology KIDNEY360 Accessing Our World From Every Angle CJASN Clinical Journal of the American Society of Nephrology

ASN Journal Disclosure Form

As per ASN journal policy, I have disclosed any financial relationships or commitments I have held in the past 36 months as included below. I have listed my Current Employer below to indicate there is a relationship requiring disclosure. If no relationship exists, my Current Employer is not listed.

D. Swinkels has nothing to disclose.

I understand that the information above will be published within the journal article, if accepted, and that failure to comply and/or to accurately and completely report the potential financial conflicts of interest could lead to the following: 1) Prior to publication, article rejection, or 2) Post-publication, sanctions ranging from, but not limited to, issuing a correction, reporting the inaccurate information to the authors' institution, banning authors from submitting work to ASN journals for varying lengths of time, and/or retraction of the published work.

Name: Dorine W. Swinkels
Manuscript ID: K360-2025-000408R1
Manuscript Title: Higher Urinary Iron Levels are Associated with Kidney Dysfunction, Tubular Damage, and Increased Mortality in Kidney Transplant Recipients
Date of Completion: June 5, 2025
Disclosure Updated Date: June 5, 2025

ASN American Society of Nephrology | JASN Journal of the American Society of Nephrology | KIDNEY360 Accessing Our World From Every Angle | CJASN Clinical Journal of the American Society of Nephrology

ASN Journal Disclosure Form

As per ASN journal policy, I have disclosed any financial relationships or commitments I have held in the past 36 months as included below. I have listed my Current Employer below to indicate there is a relationship requiring disclosure. If no relationship exists, my Current Employer is not listed.

J. Vinke reports the following:
Employer: University Medical Center Groningen; and Research Funding: Vifor Pharma.

I understand that the information above will be published within the journal article, if accepted, and that failure to comply and/or to accurately and completely report the potential financial conflicts of interest could lead to the following: 1) Prior to publication, article rejection, or 2) Post-publication, sanctions ranging from, but not limited to, issuing a correction, reporting the inaccurate information to the authors' institution, banning authors from submitting work to ASN journals for varying lengths of time, and/or retraction of the published work.

Name: Joanna Sophia Jacoline Vinke
Manuscript ID: K360-2025-000408R1
Manuscript Title: Higher Urinary Iron Levels are Associated with Kidney Dysfunction, Tubular Damage, and Increased Mortality in Kidney Transplant Recipients
Date of Completion: June 10, 2025
Disclosure Updated Date: June 10, 2025
