# Supplementary material for: Higher Urinary Iron Levels Are Associated with Kidney Dysfunction, Tubular Damage, and Increased Mortality in Kidney Transplant Recipients
Source: Kidney360. 2025 Jun 26;6(11):1970–80. doi: 10.34067/KID.0000000878 (PMC12626665; doi:10.34067/KID.0000000878)
Supplement: Supplementary file 2 [file kidney360-6-1970-s002.pdf]

Online Supplemental Material

Urinary Iron and Death in Kidney Transplant Recipients

Daan Kremer, MD[1$], Pien Rawee, MSc[1$], Tim J Knobbe, MD[1], Joanna Sophia J Vinke, MD[1], Kai Lüersen, PhD[2], David E Leaf, MD/MMSc[3], Dorine W Swinkels, MD/PhD[4,5], Martin H de Borst, MD/PhD[1], Gerald Rimbach, PhD[2*], Stephan JL Bakker, MD/PhD[1*], and Michele F Eisenga, MD/PhD [1*]

[1] Division of Nephrology, Department of Internal Medicine, University of Groningen, University Medical Center Groningen, Groningen, The Netherlands

[2] Institute of Human Nutrition and Food Science, University of Kiel, Kiel, Germany

[3] Division of Renal Medicine, Brigham and Women's Hospital, Boston, Massachusetts, USA

[4] Department of Laboratory Medicine, Radboud University Medical Center, Nijmegen, Gelderland, The Netherlands.

[5] Sanquin Blood Bank, Amsterdam, The Netherlands

[$] Contributed equally

[*] Served as co-senior authors

Corresponding authors:

Daan Kremer, M.D.

Email: d.kremer@umcg.nl

Michele F. Eisenga, M.D./PhD

Email: m.f.eisenga@umcg.nl

Table of Contents

Supplemental Figure 1: Kaplan Meier curves for graft failure and mortality

Supplemental Table 1: Iron analysis using inductively coupled plasma mass spectrometry.

Supplemental Table 2: Univariable and adjusted Cox proportional hazards analyses for the association of urinary iron concentration with graft failure and mortality, with exclusion of patients with iron overload (*i.e.*, transferrin saturation >45%; N=33)

Supplemental Table 3: Univariable and adjusted Cox proportional hazards analyses for the association of urinary iron concentration with graft failure and mortality, with exclusion of patients using iron supplementation (N=41)

Supplemental Table 4: Univariable and adjusted Cox proportional hazards analyses for the association of urinary iron concentration with graft failure and mortality, with exclusion of patients with suspected UTI (N=66)

Supplemental Table 5: Univariable and adjusted Cox proportional hazards analyses for the association of 24h urinary iron excretion (rather than concentration) with graft failure and mortality

Strobe Checklist: Checklist of items that should be included in reports of observational studies

Supplemental Figure 1 | Kaplan Meier curves for graft failure & mortality.

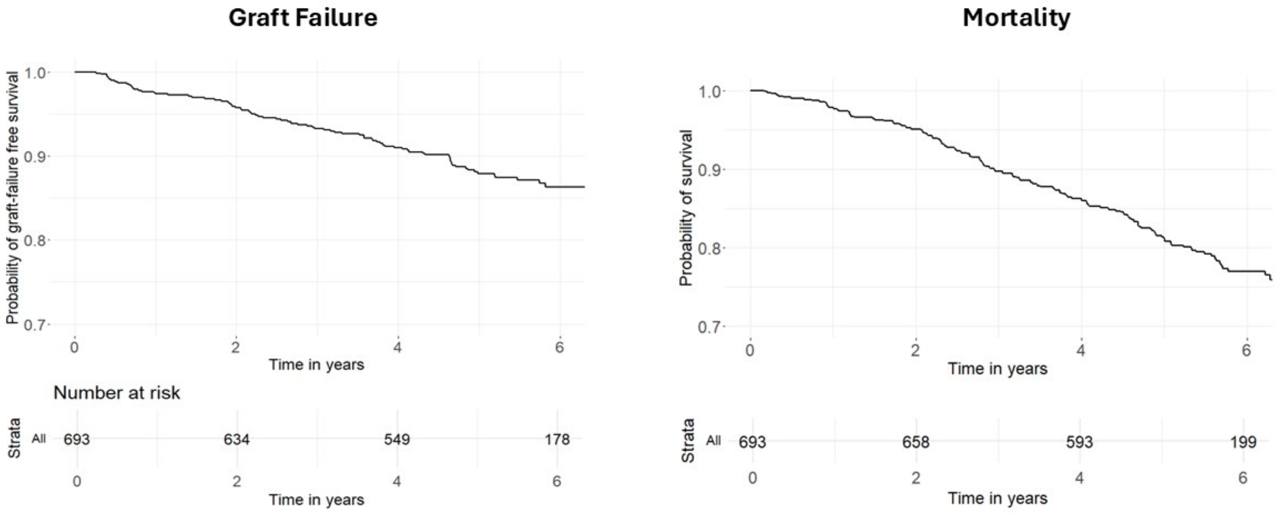

Supplemental Table 1 | Iron analysis using inductively coupled plasma mass spectrometry.

**Experimental conditions**	
**Apparatus**	ICAPQ (Thermo Fisher Scientific Waltham)
**Method**	DIN EN ISO 17294-2: 2017-01<br>Conducted by SYNLAB Analytics & Service, Jena, Germany
**Final sample dilution**	1 to 50 (2 % (v/v)) nitric acid
**Internal standard**	Rhodium (2 µg/L)
**Limit of detection (LOD)**	0.3 µg/l
**Limit of quantification (LOQ)**	1.0 µg/l
**Recovery**	100.5%
**Intra-day precision, coefficient of variation**	0.4%

Supplemental Table 2 | Univariable and adjusted Cox proportional hazards analyses for the association of urinary iron concentration with graft failure and mortality, with exclusion of patients with iron overload (*i.e.*, transferrin saturation >45%; N=33)

	Graft Failure (N_{events} = 77/660, 12%)		Mortality (N_{events} = 147/660, 22%)	
**Model**	**HR per doubling (95% CI)**	**P-value**	**HR per doubling (95% CI)**	**P-value**
Model 1	1.57 (1.30 to 1.90)	<0.001	1.29 (1.10 to 1.50)	0.001
Model 2	1.55 (1.28 to 1.89)	<0.001	1.43 (1.22 to 1.68)	<0.001
Model 3	1.31 (1.07 to 1.61)	0.010	1.37 (1.16 to 1.61)	<0.001
Model 4	1.04 (0.80 to 1.34)	0.8	1.27 (1.06 to 1.51)	0.010
Model 5	0.98 (0.75 to 1.29)	0.9	1.30 (1.10 to 1.55)	0.003
Model 6	1.15 (0.87 to 1.51)	0.3	1.29 (1.07 to 1.55)	0.008
Model 7	1.07 (0.81 to 1.42)	0.6	1.27 (1.05 to 1.54)	0.01

Model 1, univariable. **Model 2**, adjusted for age, sex, and log_2 time after transplantation. **Model 3**, adjusted for variables in model 2 + eGFR; **Model 4**, adjusted for variables in model 3 + log_2 24h urinary protein excretion; **Model 5**, adjusted for variables in model 3 + log_2 24h urinary albumin excretion; **Model 6**, adjusted for variables in model 4 + pre-emptive transplantation, history of cardiovascular disease, systolic blood pressure, donor type (living or postmortal) and history of rejection; **Model 7**, adjusted for variables in model 4 + urinary epidermal growth factor to creatinine ratio, plasma neutrophil gelatinase-associated lipocalin, 24h urinary liver-type fatty acid-binding protein, and plasma endotrophin. Addition of urinary iron concentration significantly improved the model fit for mortality ($P_{likelihood\ ratio}$=0.012) but not for graft failure ($P_{likelihood\ ratio}$=0.8). HR, hazard ratio; CI, confidence interval.

Supplemental Table 3 | Univariable and adjusted Cox proportional hazards analyses for the association of urinary iron concentration with graft failure and mortality, with exclusion of patients using iron supplementation (N=41)

	**Graft Failure (N_{events} = 71/652, 11%)**		**Mortality (N_{events} = 133/652, 20%)**	
**Model**	**HR per doubling (95% CI)**	**P-value**	**HR per doubling (95% CI)**	**P-value**
Model 1	1.66 (1.37 to 2.00)	<0.001	1.25 (1.06 to 1.47)	0.009
Model 2	1.64 (1.35 to 2.00)	<0.001	1.41 (1.19 to 1.67)	<0.001
Model 3	1.37 (1.12 to 1.69)	0.003	1.35 (1.13 to 1.60)	<0.001
Model 4	1.07 (0.83 to 1.38)	0.6	1.23 (1.02 to 1.49)	0.04
Model 5	1.02 (0.76 to 1.35)	0.9	1.26 (1.04 to 1.53)	0.02
Model 6	1.20 (0.91 to 1.59)	0.2	1.27 (1.04 to 1.55)	0.02
Model 7	1.14 (0.87 to 1.50)	0.3	1.25 (1.02 to 1.53)	0.03

Model 1, univariable. **Model 2**, adjusted for age, sex, and $\log_2$ time after transplantation. **Model 3**, adjusted for variables in model 2 + eGFR; **Model 4**, adjusted for variables in model 3 + $\log_2$ 24h urinary protein excretion; **Model 5**, adjusted for variables in model 3 + $\log_2$ 24h urinary albumin excretion; **Model 6**, adjusted for variables in model 4 + pre-emptive transplantation, history of cardiovascular disease, systolic blood pressure, donor type (living or postmortal) and history of rejection; **Model 7**, adjusted for variables in model 4 + urinary epidermal growth factor to creatinine ratio, plasma neutrophil gelatinase-associated lipocalin, 24h urinary liver-type fatty acid-binding protein, and plasma endotrophin. Addition of urinary iron concentration significantly improved the model fit for mortality ($P_{likelihood\ ratio}$=0.048) but not for graft failure ($P_{likelihood\ ratio}$=0.9). HR, hazard ratio; CI, confidence interval.

Supplemental Table 4 | Univariable and adjusted Cox proportional hazards analyses for the association of urinary iron concentration with graft failure and mortality, with exclusion of patients with suspected UTI (N=66)

	Graft Failure (N_{events} = 74/636, 12%)		Mortality (N_{events} = 136/636, 21%)	
**Model**	**HR per doubling (95% CI)**	**P-value**	**HR per doubling (95% CI)**	**P-value**
Model 1	1.70 (1.41 to 2.06)	<0.001	1.29 (1.10 to 1.52)	0.002
Model 2	1.68 (1.38 to 2.04)	<0.001	1.47 (1.24 to 1.74)	<0.001
Model 3	1.40 (1.13 to 1.73)	0.003	1.40 (1.18 to 1.67)	<0.001
Model 4	1.11 (0.86 to 1.44)	0.42	1.30 (1.08 to 1.58)	0.006
Model 5	1.06 (0.80 to 1.40)	0.68	1.35 (1.12 to 1.62)	0.002
Model 6	1.19 (0.91 to 1.55)	0.20	1.31 (1.08 to 1.59)	0.006
Model 7	1.15 (0.86 to 1.53)	0.34	1.31 (1.07 to 1.60)	0.01

Model 1, univariable. **Model 2**, adjusted for age, sex, and log_2 time after transplantation. **Model 3**, adjusted for variables in model 2 + eGFR; **Model 4**, adjusted for variables in model 3 + log_2 24h urinary protein excretion; **Model 5**, adjusted for variables in model 3 + log_2 24h urinary albumin excretion; **Model 6**, adjusted for variables in model 4 + pre-emptive transplantation, history of cardiovascular disease, systolic blood pressure, donor type (living or postmortal) and history of rejection; **Model 7**, adjusted for variables in model 4 + urinary epidermal growth factor to creatinine ratio, plasma neutrophil gelatinase-associated lipocalin, 24h urinary liver-type fatty acid-binding protein, and plasma endotrophin. Addition of urinary iron concentration significantly improved the model fit for mortality ($P_{likelihood\ ratio}$=0.008) but not for graft failure ($P_{likelihood\ ratio}$=0.42). HR, hazard ratio; CI, confidence interval.

Supplemental Table 5 | Univariable and adjusted Cox proportional hazards analyses for the association of 24h urinary iron excretion (rather than concentration) with graft failure and mortality

	Graft Failure (N_{events} = 83/693, 12%)		Mortality (N_{events} = 150/693, 22%)	
**Model**	**HR per SD (95% CI)**	**P-value**	**HR per SD (95% CI)**	**P-value**
Model 1	1.15 (1.02 to 1.29)	0.02	1.14 (1.04 to 1.25)	0.006
Model 2	1.15 (1.01 to 1.29)	0.03	1.18 (1.09 to 1.28)	<0.001
Model 3	1.06 (0.92 to 1.23)	0.4	1.15 (1.06 to 1.26)	0.002
Model 4	0.93 (0.73 to 1.19)	0.5	1.13 (1.03 to 1.24)	0.01
Model 5	1.02 (0.79 to 1.32)	0.9	1.33 (1.12 to 1.58)	0.001
Model 6	1.19 (0.93 to 1.54)	0.16	1.17 (1.06 to 1.30)	0.003
Model 7	0.98 (0.76 to 1.27)	0.9	1.15 (1.04 to 1.26)	0.005

Model 1, adjusted for 24h urinary volume. **Model 2**, adjusted for model 1 + age, sex, and $\log_2$ time after transplantation. **Model 3**, adjusted for variables in model 2 + eGFR; **Model 4**, adjusted for variables in model 3 + $\log_2$ 24h urinary protein excretion; **Model 5**, adjusted for variables in model 3 + $\log_2$ 24h urinary albumin excretion; **Model 6**, adjusted for variables in model 4 + pre-emptive transplantation, history of cardiovascular disease, systolic blood pressure, donor type (living or postmortal) and history of rejection; **Model 7**, adjusted for variables in model 4 + urinary epidermal growth factor to creatinine ratio, plasma neutrophil gelatinase-associated lipocalin, 24h urinary liver-type fatty acid-binding protein, and plasma endotrophin. Addition of 24h urinary iron excretion significantly improved the model fit for mortality ($P_{likelihood\ ratio}$=0.006) but not for graft failure ($P_{likelihood\ ratio}$=0.1). HR, hazard ratio; CI, confidence interval.

STROBE Statement—checklist of items that should be included in reports of observational studies

	Item No.	Recommendation	Page No.
**Title and abstract**	1	(*a*) Indicate the study's design with a commonly used term in the title or the abstract	2 (abstract)
		(*b*) Provide in the abstract an informative and balanced summary of what was done and what was found	2-3 (abstract)
**Introduction**			
Background/rationale	2	Explain the scientific background and rationale for the investigation being reported	4 (Introduction, paragraph 2)
Objectives	3	State specific objectives, including any prespecified hypotheses	4 (Introduction, paragraph 3)
**Methods**			
Study design	4	Present key elements of study design early in the paper	5-6 (Methods, "Study population" and "Outcome definitions")
Setting	5	Describe the setting, locations, and relevant dates, including periods of recruitment, exposure, follow-up, and data collection	5 (Methods, "Study population")
Participants	6	(*a*) Give the eligibility criteria, and the sources and methods of selection of participants. Describe methods of follow-up	5 (Methods, "Study population")<br><br>Figure 1
		(*b*) For matched studies, give matching criteria and number of exposed and unexposed	N.A
Variables	7	Clearly define all outcomes, exposures, predictors, potential confounders, and effect modifiers. Give diagnostic criteria, if applicable	6 (Methods, "Outcome definitions")<br><br>5 (Methods, "Study population")

			7-8 (Methods, “Statistical analyses”)
Data sources/ measurement	8*	For each variable of interest, give sources of data and details of methods of assessment (measurement). Describe comparability of assessment methods if there is more than one group	6-7 (Methods, “Biochemical analyses”)
Bias	9	Describe any efforts to address potential sources of bias	7-8 (Methods, “Statistical analyses”)
Study size	10	Explain how the study size was arrived at	5 (Methods, “Study population”)<br>Figure 1

Continued on next page

Quantitative variables	11	Explain how quantitative variables were handled in the analyses. If applicable, describe which groupings were chosen and why	7 (Methods, "Statistical analyses")
Statistical methods	12	(*a*) Describe all statistical methods, including those used to control for confounding	7-8 (Methods, "Statistical analyses")
		(*b*) Describe any methods used to examine subgroups and interactions	7-8 (Methods, "Statistical analyses")
		(*c*) Explain how missing data were addressed	8 (Methods, "Statistical analyses")
		(*d*) *Cohort study*—If applicable, explain how loss to follow-up was addressed	N.A.
		(*e*) Describe any sensitivity analyses	8 (Methods, "Statistical analyses")
Participants	13*	(a) Report numbers of individuals at each stage of study—eg numbers potentially eligible, examined for eligibility, confirmed eligible, included in the study, completing follow-up, and analysed	Figure 1
		(b) Give reasons for non-participation at each stage	Figure 1
		(c) Consider use of a flow diagram	Figure 1
Descriptive data	14*	(a) Give characteristics of study participants (eg demographic, clinical, social) and information on exposures and potential confounders	20-22 (Table 1)
		(b) Indicate number of participants with missing data for each variable of interest	22 (Description under Table 1)
		(c) *Cohort study*—Summarise follow-up time (eg, average and total amount)	10 (Results, "Urinary iron, graft failure and death")
Outcome data	15*	*Cohort study*—Report numbers of outcome events or summary measures over time	10 (Results, "Urinary iron, graft failure and death")

		Case-control study—Report numbers in each exposure category, or summary measures of exposure	N.A.
		Cross-sectional study—Report numbers of outcome events or summary measures	N.A.
Main results	16	(*a*) Give unadjusted estimates and, if applicable, confounder-adjusted estimates and their precision (eg, 95% confidence interval). Make clear which confounders were adjusted for and why they were included	20-22 (Table 1)<br><br>23 (Table 2)
		(*b*) Report category boundaries when continuous variables were categorized	N.A.
		(*c*) If relevant, consider translating estimates of relative risk into absolute risk for a meaningful time period	N.A.

Continued on next page

Other analyses	17	Report other analyses done—eg analyses of subgroups and interactions, and sensitivity analyses	24 (Table 3)<br><br>Figure 2 and 3<br><br>Table S2-S5
Key results	18	Summarise key results with reference to study objectives	11 (Discussion, paragraph 1)
Limitations	19	Discuss limitations of the study, taking into account sources of potential bias or imprecision. Discuss both direction and magnitude of any potential bias	14 -15 (Discussion, paragraph 9)
Interpretation	20	Give a cautious overall interpretation of results considering objectives, limitations, multiplicity of analyses, results from similar studies, and other relevant evidence	15 (Discussion, paragraph 10, i.e. last)
Generalisability	21	Discuss the generalisability (external validity) of the study results	15 (Discussion, paragraph 9)
**Other information**			
Funding	22	Give the source of funding and the role of the funders for the present study and, if applicable, for the original study on which the present article is based	16 (Funding)

*Give information separately for cases and controls in case-control studies and, if applicable, for exposed and unexposed groups in cohort and cross-sectional studies.
